# Rhn1, a Nuclear Protein, Is Required for Suppression of Meiotic mRNAs in Mitotically Dividing Fission Yeast

**DOI:** 10.1371/journal.pone.0042962

**Published:** 2012-08-17

**Authors:** Tomoyasu Sugiyama, Rie Sugioka-Sugiyama, Kazumasa Hada, Ryusuke Niwa

**Affiliations:** 1 Graduate School of Life and Environmental Sciences, University of Tsukuba, Tsukuba, Ibaraki, Japan; 2 Initiative for the Promotion of Young Scientists' Independent Research, University of Tsukuba, Tsukuba, Ibaraki, Japan; 3 Precursory Research for Embryonic Science and Technology (PRESTO), Japan Science and Technology Agency (JST), Kawaguchi, Saitama, Japan; Newcastle University, United Kingdom

## Abstract

In the fission yeast *Schizosaccharomyces pombe*, many meiotic mRNAs are transcribed during mitosis and meiosis and selectively eliminated in mitotic cells. However, this pathway for mRNA decay, called the determinant of selective removal (DSR)-Mmi1 system, targets only some of the numerous meiotic mRNAs that are transcribed in mitotic cells. Here we describe Rhn1, a nuclear protein involved in meiotic mRNA suppression in vegetative fission yeast. Rhn1 is homologous to budding yeast Rtt103 and localizes to one or a few discrete nuclear dots in growing vegetative cells. Rhn1 colocalizes with a pre-mRNA 3′-end processing factor, Pcf11, and with the 5′–3′ exoribonuclease, Dhp1; moreover, Rhn1 coimmunoprecipitates with Pcf11. Loss of *rhn1* results in elevated sensitivity to high temperature, to thiabendazole (TBZ), and to UV. Interestingly, meiotic mRNAs—including *moa1^+^, mcp5^+^, and mug96^+^*—accumulate in mitotic *rhn1*Δ cells. Accumulation of meiotic mRNAs also occurs in strains lacking Lsk1, a kinase that phosphorylates serine 2 (Ser-2) in the C-terminal domain (CTD) of RNA polymerase II (Pol II), and in strains lacking Sen1, an ATP-dependent 5′–3′ RNA/DNA helicase: notably, both Lsk1 and Sen1 have been implicated in termination of Pol II-dependent transcription. Furthermore, RNAi knockdown of *cids-2*, a *Caenorhabditis elegans* ortholog of *rhn1* ^+^, leads to elevated expression of a germline-specific gene, *pgl-1*, in somatic cells. These results indicate that Rhn1 contributes to the suppression of meiotic mRNAs in vegetative fission yeast and that the mechanism by which Rhn1 downregulates germline-specific transcripts may be conserved in unicellular and multicellular organisms.

## Introduction

Many kinds of compartments, collectively designated nuclear bodies, are present in nuclei of eukaryotic cells [1], [2]. Nuclear bodies comprise proteins and RNAs and are believed to have important roles in various nuclear functions, including RNA processing, snRNP assembly, and transcription. For example, nuclear speckles are thought to function in the assembly and storage of pre-mRNA splicing factors [3]. Similarly, paraspeckles participate in the regulation of gene expression by retaining mRNAs that carry inverted repeats and are extensively A-to-I edited; moreover, these paraspeckles may be useful as a structural marker in discriminating between pluripotent and differentiated cells [4]. Apparently, many nuclear proteins involved in specific tasks are assembled into discrete nuclear compartments to perform their respective functions efficiently. However, the mechanisms of nuclear body assembly and the exact function of each type of these structures are not fully understood [5].

RNA metabolism is an important activity in both prokaryotes and eukaryotes. RNA degradation pathways are basically classified as follows: (1) RNA processing of primary transcripts to produce mature mRNAs; (2) regulated turnover of mRNAs and non-coding RNAs to modulate gene expression; and (3) quality control systems that remove defective RNAs and RNA-protein complexes from cells [6], [7]. In eukaryotic cells, these tasks require many exoribonuclease, endoribonucleases, RNA helicases, poly(A) polymerases, chaperones, and small RNAs [6], [7].

Recent studies have highlighted the fact that several mRNA degradation factors play key roles in other cellular mechanisms, including termination of RNA polymerase II (Pol II) transcription [7]. In the budding yeast *Saccharomyces cerevisiae*, serine 2 (Ser-2) phosphorylation of heptamer repeat sequences in the C-terminal domain (CTD) of Pol II facilitates *in vitro* interactions between this CTD and Rtt103, a protein with a CTD-interacting domain (CID), and between this CID and Pcf11, a pre-mRNA 3′-end processing factor [8], [9]. Rtt103 also associates with Rat1, a 5′–3′ exoribonuclease, and with Rai1, a Rat1-interacting protein [8]. Rat1 degrades transcripts with uncapped 5′-ends generated by pre-mRNA cleavage, and it eventually promotes the dissociation of Pol II from template DNAs [10]. Thus Rat1, Rai1, Rtt103, and polyadenylation factors contribute to, at least in part, termination of Pol II-dependent transcription. This termination mechanism has been designated the torpedo model and is apparently conserved in human because RNAi knockdown of Xrn2, a Rat1 homolog in higher eukaryotes, results in a decrease in transcriptional termination efficiency [10], [11].

In the fission yeast *Schizosaccharomyces pombe*, selective mRNA decay, as well as transcriptional control, has emerged as a fundamental regulatory mechanism in the control of meiotic gene expression. Many genes that are upregulated during meiosis are transcribed even in vegetative cells, but these meiotic transcripts are subjected to mRNA degradation in the vegetative cells [12]–[14]. This mRNA degradation pathway requires specific sequences (designated determinant of selective removal (DSR)), an RNA-binding protein (Mmi1), a 3′-end processing factor (Rna15), the canonical poly(A) polymerase (Pla1), a nuclear poly(A)-binding protein (Pab2), a zinc-finger protein (Red1), and the nuclear RNase complex exosome; taken together, these requirements indicated that the DSR system is a poly(A)-dependent mRNA degradation pathway [12], [14]–[18]. In addition, DSR-mediated mRNA elimination may be inactivated during meiosis, and inactivation of the DSR pathway may allow meiotic mRNAs to be translated [12]. Thus, in fission yeast, the untimely expression of meiosis-specific mRNAs is believed to be prevented via this DSR-mediated mRNA decay. However, it is still unclear whether transcriptional control and DSR-mediated selective mRNA elimination are sufficient for regulating all of the genes that are upregulated during meiosis, and there may be additional mechanism(s) necessary for the control of meiotic gene expression.

We used protein localization methods to identify several factors that localize to nuclear or cytoplasmic foci in fission yeast. We previously reported that one such protein, Red1, localizes to cleavage bodies and promotes selective elimination of meiosis-specific mRNAs in vegetative fission yeast [15]. Here, we report that Rhn1, another factor that localizes to nuclear dots, contributes to the suppression of meiotic genes in vegetative fission yeast. Characterization of Rhn1 should help to elucidate the complex mechanisms that suppress untimely expression of numerous meiotic mRNAs in vegetative fission yeast, as well as in somatic cells of higher eukaryotes, including the nematode *Caenorhabditis elegans*.

## Results and Discussion

### Rhn1 localizes to the nucleus and forms nuclear dots

**Figure 1:**
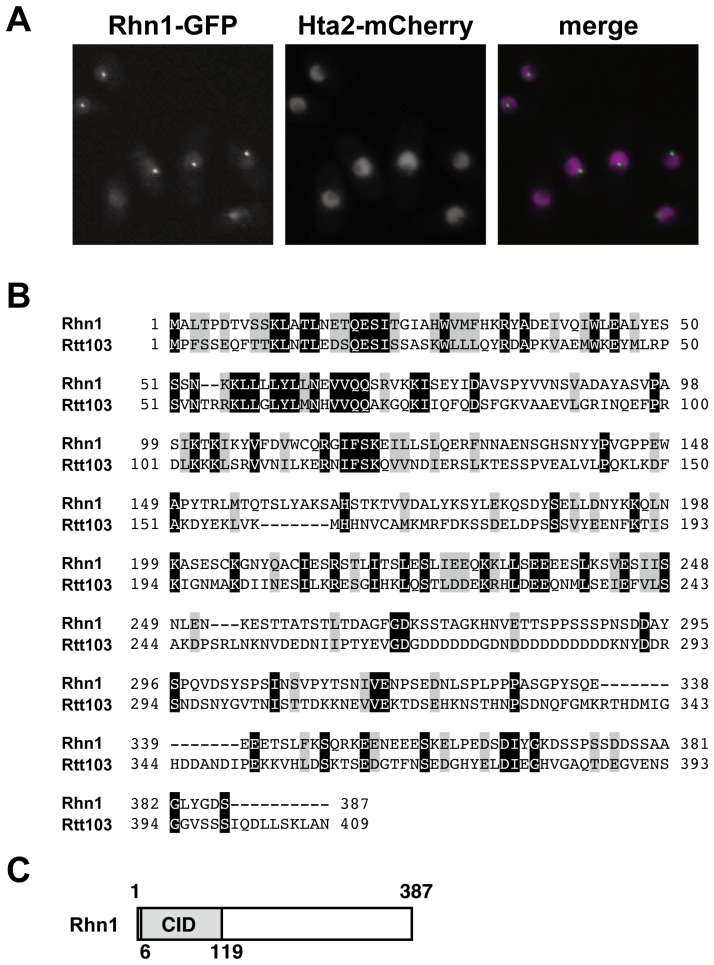
Rhn1 is a nuclear protein and is homologous to budding yeast Rtt103. (A) Rhn1 localizes to the nucleus. Exponentially growing cells expressing both Rhn1-GFP and Hta2 (histone H2A)-mCherry [20], [21] in YEA cultures were observed using fluorescent microscopy. Representative images are shown. (B) Amino acid sequence alignments of Rhn1 and its budding yeast homolog Rtt103. ClustalW2 (http://www.ebi.ac.uk/Tools/clustalw2/index.html) was used to align these sequences. Black and gray shadings represent identical and similar amino acids, respectively. (C) Schematic representation of Rhn1. CID, CTD-interacting domain.

To identify novel factors that regulate RNA metabolism in fission yeast, we utilized a subcellular localization database [19] and selected candidate proteins that localize to the nucleus. We then constructed fission yeast strains that each expressed one candidate protein as a fusion protein that had either a carboxy-terminal tdTomato tag, an mCherry tag, or a GFP tag [20], [21]. Among these candidate proteins, the previously uncharacterized protein encoded by *SPBC337.03* formed one or a few distinct dots under vegetative growth conditions (Figure 1A). We also found that the signal from the SPBC337.03-GFP fusion protein was distributed evenly throughout nuclei of vegetative cells (Figure 1A). *SPBC337.03* was predicted to encode a 387 amino acid protein that had homology to budding yeast Rtt103 (Figure 1B); we named this gene *rhn1* ^+^ (Rtt103 homolog that localizes to the nucleus 1). *S. cerevisiae* Rtt103 has a CID (CTD-interacting domain) that associates with the CTD of Pol II, and was originally identified as a regulator of Ty1 transposition [22]. Rhn1, like Rtt103, contained a CID (Figure 1C); this finding indicated that Rhn1 may interact with Pol II.

### *rhn1*Δ cells are sensitive to high temperature, TBZ, and UV

**Figure 2:**
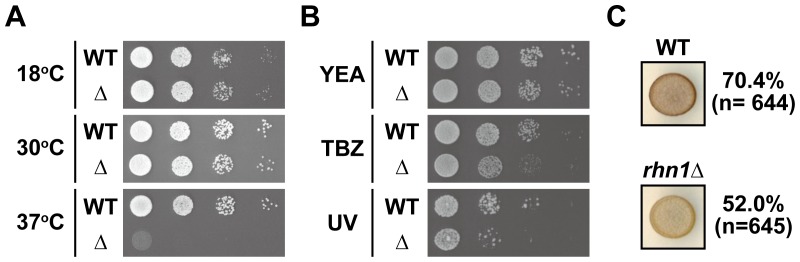
Phenotypic characterization of *rhn1*Δ cells. (A) *rhn1*Δ cells were sensitive to high temperature. Serial dilutions of wild-type (WT) and *rhn1*Δ (Δ) cells were spotted on YEA plates, and the plates were then incubated at the indicated temperatures for 2 to 6 days. (B) Sensitivity to thiabendazole (TBZ) and UV in *rhn1*Δ cells. Serial dilutions of wild-type (WT) and *rhn1*Δ (Δ) cells were spotted on YEA plates (YEA), YEA plates supplemented with 16 µg/ml of TBZ (TBZ), or YEA plates exposed to 160 J/m^2^ of UV (UV) after spotting of cells; cells were then incubated at 30°C for 2 to 3 days. (C) The effect of *rhn1*Δ on mating. Wild-type (WT) and *rhn1*Δ cells were grown on PMG plates supplemented with thiamine at 26°C for 3 days and then stained with iodine vapors. Representative images are shown. The mating efficiencies of wild-type and *rhn1*Δ cells are also shown. “n” indicates the number of cells counted.

To characterize the function of Rhn1, we constructed an *rhn1* deletion strain (*rhn1*Δ) and analyzed the phenotypes of *rhn1*Δ cells. We observed neither severe growth defects nor growth retardation of *rhn1*Δ cells at 18 or 30°C; however, *rhn1*Δ cells did not at all grow at 37°C (Figure 2A); this finding indicated that Rhn1 was necessary for growth at high temperature. Analyses of serial dilutions of cell cultures also revealed that *rhn1*Δ cells were more sensitive to thiabendazole (TBZ), a microtubule-destabilizing drug, and UV than were wild-type cells (Figure 2B); therefore, Rhn1 may function to maintain genomic stability.

### *rhn1*Δ cells are defective in mating

Fission yeast cells that have Ser-2 to alanine substitutions in the Pol II CTD are sterile [23]–[25], and Rtt103 binds to phosphorylated Ser-2 of CTD [8]; therefore, we hypothesized that Rhn1 had a role in mating. To test this hypothesis, we compared sporulation of wild-type cells and *rhn1*Δ cells and found that iodine staining, which selectively labels sporulated cells, was less intense in *rhn1*Δ cells than in wild-type cells (Figure 2C). Consistent with this result, mating efficiency was lower in *rhn1*Δ cells than in wild-type cells (70.4% vs 52.0%) (Figure 2C). The reduction in iodine staining may have been due to impaired mating in *rhn1*Δ cells, and it indicated that Rhn1 was required for efficient mating.

### Rhn1 colocalizes and interacts with the pre-mRNA 3′-end cleavage factor Pcf11

**Figure 3:**
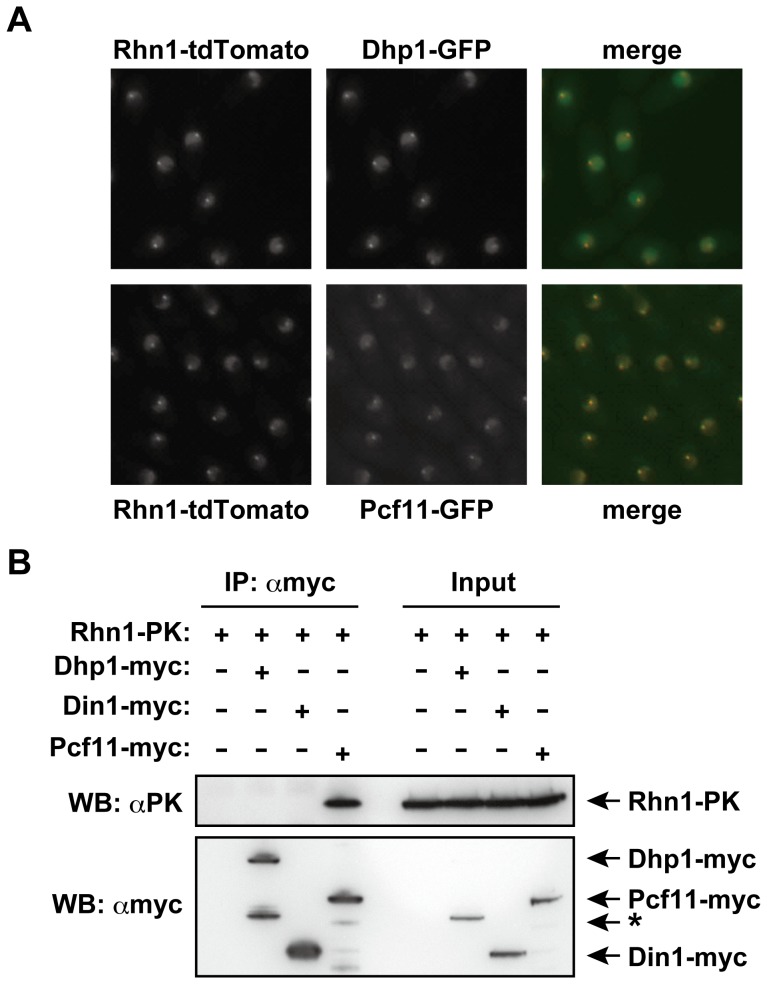
Rhn1 colocalizes and interacts with the pre-mRNA 3′-end cleavage factor Pcf11. (A) Colocalization of Rhn1 with Dhp1 and Pcf11 in vegetative cells. Exponentially growing cells expressing Rhn1-tdTomato and Dhp1-GFP or Rhn1-tdTomato and Pcf11-GFP in YEA cultures were subjected to fluorescence microscopic analyses. Representative images are shown. (B) Rhn1 interacts with Pcf11. Total cell lysates prepared from strains expressing Rhn1-PK, Rhn1-PK and Dhp1-myc, Rhn1-PK and Din1-myc, or Rhn1-PK and Pcf11-myc were subjected to immunoprecipitation using anti-myc; immnoprecipitates and inputs were analyzed on Western blots. The asterisk indicates the degradation product of Dhp1-myc protein. Note that, because of its low abundance, full-length Dhp1-myc was only visible when it was concentrated by immunoprecipitation.

In *S. cerevisiae*, Rtt103 physically associates with Rat1, Rai1, and Pcf11 [8]. Based on this, we hypothesized that Rhn1 associates with Dhp1 (a 5′–3′ exoribonuclease) and Din1 (a Dhp1-binding protein), the fission yeast homologs of Rat1 and Rai1 [26], [27], respectively, and with Pcf11. To test this hypothesis, we first examined whether Rhn1 colocalized with Dhp1, Pcf11, or both; as expected, Rhn1 colocalized with Dhp1 and with Pcf11 (Figure 3A). Interestingly, immunoprecipitation assays revealed that Rhn1 coimmunoprecipitated with Pcf11, but not with Dhp1 or Din1 (Figure 3B), indicating that Rhn1 stably associated with Pcf11 in vegetative cells.

### Rhn1 contributes to the suppression of meiotic mRNAs during mitosis

**Figure 4:**
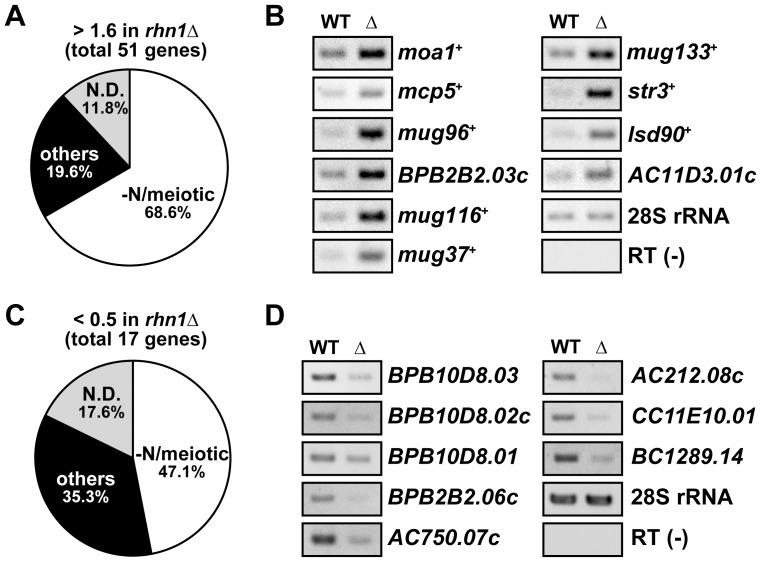
Expression profiling of vegetative *rhn1*Δ cells. (A) A pie chart of the classification of mRNAs accumulated in vegetatively growing *rhn1*Δ cells. Expression analyses using a microarray technique demonstrated that 51 genes were upregulated (>1.6-fold) in vegetatively growing *rhn1*Δ cells, and subsequent analysis revealed that 35 of these 51 genes (68.6%) were previously reported to be genes upregulated in response to nitrogen starvation, pheromone treatment, or during meiosis (-N/meiosis). In addition, 10 genes were non-meiotic genes (others) and 6 genes were not known to be meiosis-related (N.D.). (B) Total RNAs isolated from wild-type (WT) and *rhn1*Δ (Δ) cells were subjected to RT-PCR analyses to confirm the results of the expression profiling. The 28S rRNA was used as a control. RT (-), no reverse transcription. (C) A pie chart of the classification of mRNAs downregulated (<0.5-fold) in *rhn1*Δ cells. Total 17 genes were identified and classified into three categories as described in (A). (D) RT-PCR of several mRNAs downregulated in *rhn1*Δ cells. RT-PCR was done as described in (B).

We next performed transcriptional expression profiling of vegetative *rhn1*Δ cells to investigate the role(s) of Rhn1 in controlling steady-state levels of mRNAs. Using a microarray technique, we identified 51 mRNAs with steady-state levels that were increased more than 1.6-fold in *rhn1*Δ cells relative to wild-type cells (Figure 4A and Table S1). The majority of these mRNAs, 35 out of 51 (68.6%), were previously reported to be meiotic mRNAs that were upregulated during meiosis [28], [29]. Among the 51 mRNAs, 10 (19.6%) were non-meiotic mRNAs, and 6 (11.8%) have not yet been as meiosis-related (Figure 4A and Table S1). We also identified 17 mRNAs with steady-state levels that were lower (<0.5-fold reduction) in *rhn1*Δ than in wild-type cells (Figure 4C and Table S2). Many (14 out of 17 mRNAs) of these downregulated genes were located in subtelomeric regions, and 8 out of 17 mRNAs were classified as meiotic genes. Some of these array results were confirmed in conventional RT-PCR experiments (Figure 4B and D). These analyses indicated that Rhn1 functions in vegetative cells predominantly to downregulate meiosis-related genes and to contribute to the maintenance of expression of several subtelomeric genes.

### Rhn1 is dispensable for DSR-mediated mRNA elimination

**Figure 5:**
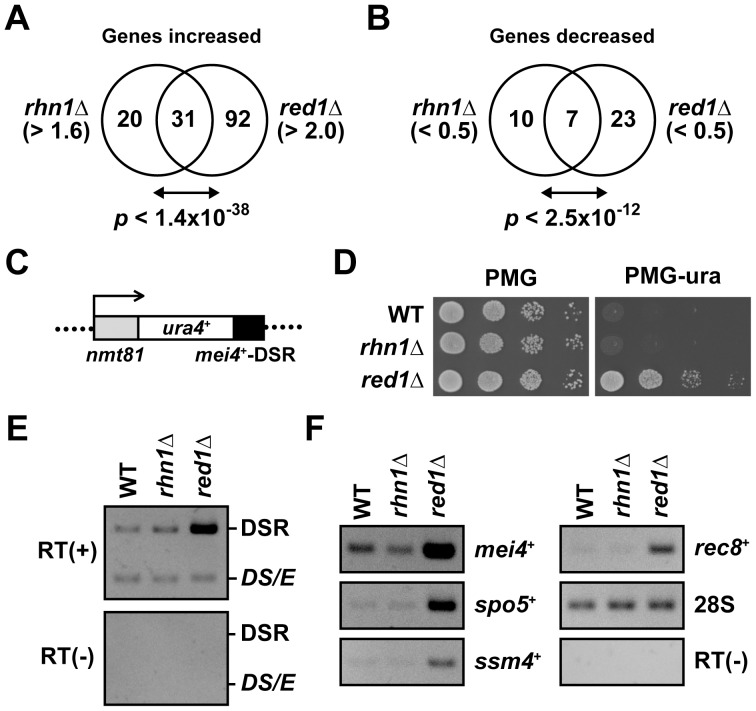
Rhn1 is dispensable for DSR-mediated mRNA elimination. (A and B) Venn diagrams of Rhn1-regulated genes and Red1-regulated genes. The genes upregulated in *rhn1*Δ (>1.6-fold) and *red1*Δ (>2-fold) strains (A) and downregulated (<0.5-fold) in *rhn1*Δ and *red1*Δ strains (B). We examined the expression of 4977 genes for these comparisons. We caluculated *p*-values to assess the statistical significance of the overlap between these groups; these *p*-values are shown under the respective diagrams. (C) Schematic representation of the *ura4* ^+^-DSR construct. The DSR region derived from *mei4* ^+^ was fused to the *ura4* ^+^ gene, and transcription of the *ura4* ^+^-DSR mRNA was driven by the thiamine-repressive *nmt81* promoter. This construct was integrated into the *lys1* ^+^ locus. (D) The effect of *ura4* ^+^-DSR mRNA elimination in *rhn1*Δ cells. Serial dilutions of wild-type (WT), *rhn1*Δ, and *red1*Δ cells carrying the *ura4* ^+^-DSR were spotted onto minimal media plates without thiamine (PMG) and onto minimal media plates lacking uracil and thiamine (PMG-ura); the plates were then incubated at 30°C for 2 to 3 days. (E) No marked accumulation of *ura4* ^+^-DSR transcripts was evident in *rhn1*Δ cells. Total RNA samples were prepared from wild-type (WT), *rhn1*Δ, or *red1*Δ cells that carries both *ura4* ^+^-DSR (DSR) and the *ura4* ^+^ minigene (*ura4DS/E* (*DS/E*)) grown in PMG media without thiamine. The levels of *ura4* ^+^-DSR and *ura4DS/E* mRNAs were determined by RT-PCR. The *ura4DS/E* transcript was used as an internal control. RT (-), no reverse transcription. (F) The steady-state levels of *mei4* ^+^, *spo5* ^+^, *ssm4* ^+^, and *rec8* ^+^ mRNAs were not higher in mitotic *rhn1*Δ cells than in wild-type cells. The total RNA samples used in (E) were also subjected to RT-PCR analyses. The 28S rRNA (28S) was used as a control. RT (-), no reverse transcription.

Meiotic mRNAs such as *mei4* ^+^ and *rec8* ^+^ are transcribed, but are then subjected to DSR-dependent mRNA degradation, in mitotically dividing cells. This degradation system requires the DSR degradation sequences (UUAAAC/UCAAAC) and several protein factors including Mmi1, Red1, Pab2, and Dhp1 [12]–[18]. Considering that some of the Red1/Mmi1-target mRNAs (e.g. *moa1* ^+^, *mug96* ^+^, and *mcp5* ^+^) accumulated in *rhn1*Δ (Figure 4A and Table S1) and that Rhn1 colocalized with Dhp1 (Figure 3A), Rhn1 might be involved in DSR-dependent mRNA decay. To assess this possibility, we first compared Rhn1-regulated genes (those genes that were upregulated or downregulated in *rhn1*Δ cells relative to wild-type cells) with Red1-regulated genes [15]; we found that the set of Rhn1-regulated genes overlapped with that of Red1-regulated genes. Specifically, 31 of 51 mRNAs (61.0%) upregulated in *rhn1*Δ cells were also upregulated in *red1*Δ cells (*p*<1.4×10^−38^) (Figure 5A), while 7 of 17 mRNAs (41.2%) downregulated in *rhn1*Δ cells were also downregulated in *red1*Δ cells (*p*<2.5×10^−12^) (Figure 5B). These results indicated that Rhn1 and Red1 suppress or promote the expression of common target mRNAs in vegetative cells and that the two proteins work in a parallel or in overlapping pathways.

Although Rhn1 and Red1 share target mRNAs, the mRNA profiles of *rhn1*Δ cells indicated that Rhn1 did not target four representative DSR-containing mRNAs, *mei4* ^+^, *rec8* ^+^, *ssm4* ^+^, and *spo5* ^+^ (Table S1). We then examined whether Rhn1 was essential for DSR-dependent RNA elimination. We introduced the *ura4* ^+^-DSR marker into wild-type and *rhn1*Δ cells (Figure 5C); this marker comprises the thiamine-repressive *nmt81* promoter, the *ura4* ^+^ open reading frame, and the DSR region of *mei4* ^+^ [12]. Wild-type and *rhn1*Δ cells carrying this marker did not grow on uracil-deficient (PMG-ura) plates; in contrast, *red1*Δ cells with the ura4^+^-DSR grew well on PMG-ura plates (Figure 5D), indicating that *ura4* ^+^-DSR was stable in *red1*Δ, but not in wild-type or *rhn1*Δ cells. This conclusion was confirmed by RT-PCR analyses; *ura4* ^+^-DSR mRNA accumulated only in *red1*Δ cells, not in wild-type or *rhn1*Δ cells (Figure 5E). Moreover, vegetative *rhn1*Δ cells did not accumulate four DSR-containing meiotic mRNAs, *mei4* ^+^, *spo5* ^+^, *ssm4* ^+^, and *rec8* ^+^ (Figure 5F), indicating that Rhn1 was not essential for DSR-dependent meiotic mRNA degradation.

### Lsk1, a subunit of Pol II CTD Ser-2 kinase, also suppresses meiotic mRNAs and genetically interacts with Rhn1

**Figure 6:**
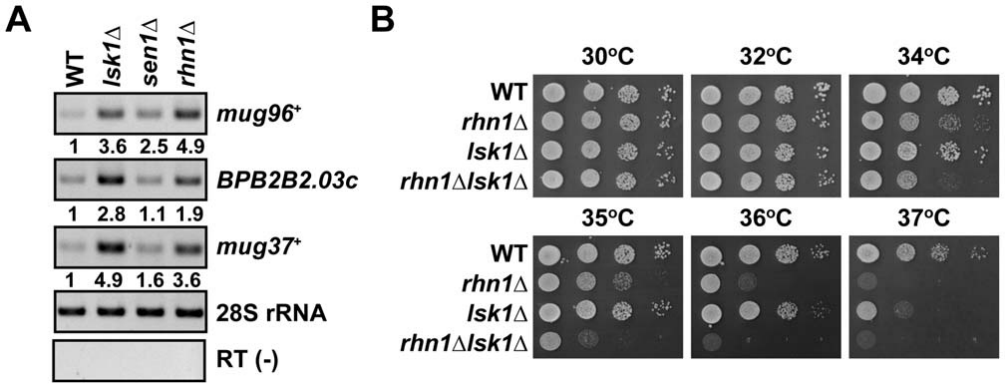
Rhn1, Lsk1, and Sen1 contribute to the suppression of meiotic mRNAs in vegetative cells. (A) The effect of an *lsk1* or *sen1* deletion on meiotic mRNA levels in vegetative cells. Total RNA isolated from wild-type (WT), *lsk1*Δ, *sen1*Δ, or *rhn1*Δ cells was subjected to RT-PCR analyses. The 28S rRNA was used as an internal control. The fold increases relative to wild-type cells are shown below each lane. RT (-), no reverse transcription. (B) Synthetic growth defect of *rhn1*Δ*lsk1*Δ cells. Serial dilutions of wild-type (WT), *rhn1*Δ, *lsk1*Δ, or *rhn1*Δ*lsk1*Δ cells were spotted onto YEA plates, and the plates were then incubated at the indicated temperatures for 3 to 4 days.

*S. cerevisiae* Rtt103 binds to the Pol II CTD, which is phosphorylated at Ser-2 *in vitro* [8]. Lsk1 is a subunit of Pol II CTD Ser-2 kinase that plays a crucial role in proper meiotic processes [23], [24], [30]. Therefore, we hypothesized that Lsk1, like Rhn1, might play a role in the suppression of meiotic mRNAs during mitosis. RT-PCR analyses showed that mitotic *lsk1*Δ cells accumulated elevated levels of meiotic mRNAs, including *mug96* ^+^ and *mug37* ^+^ (Figure 6A). We also tested whether Sen1, an ATP-dependent 5′–3′ RNA/DNA helicase [31], contributed to the suppression of meiotic mRNAs. The levels of *mug96* ^+^ transcripts, but not *SPBPB2B2.03c* or *mug37* ^+^, transcripts significantly increased in mitotic *sen1*Δ cells (Figure 6A). Our results also indicated that some of meiotic mRNAs that accumulated in mitotic *rhn1*Δ cells did not accumulate markedly in mitotic *lsk1*Δ cells or mitotic *sen1*Δ cells (Figure 6A and data not shown). We next tested for a genetic interaction between Rhn1 and Lsk1 to examine the relationship between the two proteins. We observed a synthetic growth defect at high temperatures (34 to 36°C) in *rhn1*Δ*lsk1*Δ cells (Figure 6B). Taken together, these analyses indicated that Rhn1, Lsk1, and Sen1 mediated downregulation of several meiotic mRNAs in overlapping or parallel pathways during mitosis.

About 2000 genes are upregulated during nitrogen starvation and meiotic processes [28]. Mmi1, Red1, and Pab2 apparently target only a fraction of these meiosis-related genes for selective elimination in mitosis [12], [14], [15], [17]. Therefore, we believe that there are at least four mechanisms that control proper meiotic mRNA expression and that these mechanisms are as follows: (1) transcriptional activation by Ste11 during meiosis [32]; (2) mRNA degradation by Red1, Mmi1, and other players in vegetative cells [33]; (3) transcriptional repression by Fkh2-mediated antisense transcription in vegetative cells [34]; and (4) a novel mechanism regulated by factors required for Pol II transcription termination pathways in vegetative cells (this study). The diversity of these mechanisms illustrates the complexity of meiotic mRNA regulation in fission yeast.

### Rhn1 promotes *ste11* ^+^ expression in response to nitrogen starvation

**Figure 7:**
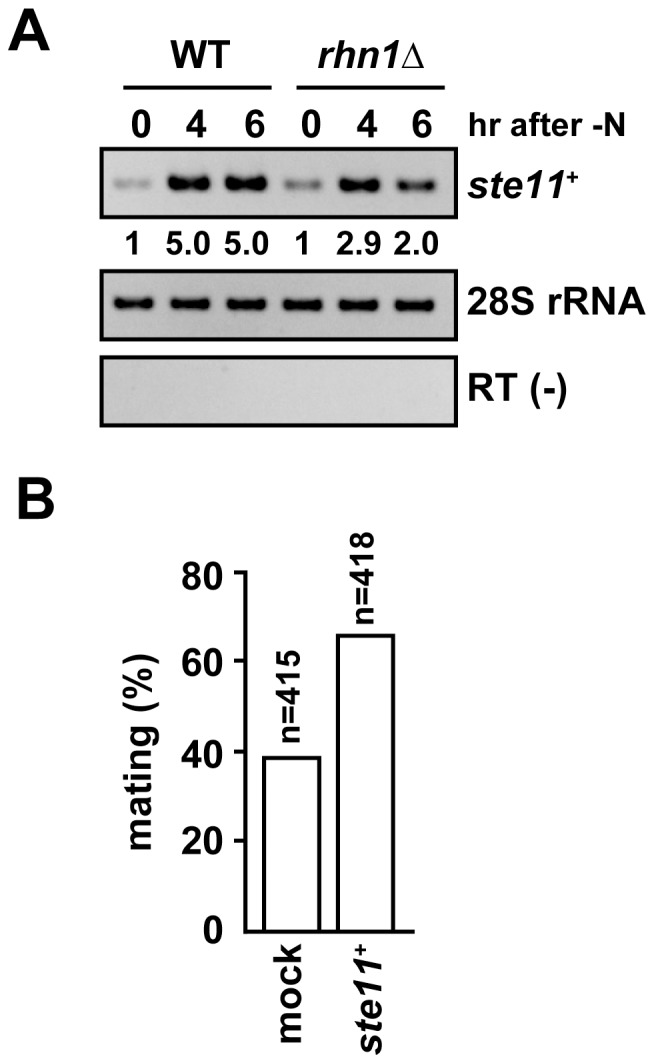
Rhn1 is essential for proper Ste11 expression. (A) *ste11* ^+^ induction after nitrogen starvation was delayed in *rhn1*Δ cells. Wild-type and *rhn1*Δ cells were grown to the mid-log phase in YEA at 30°C, washed with PMG-N, and then cultured in PMG-N at 26°C. RNA samples were collected at the indicated time points. The fold increases relative to wild-type cells are shown below each lane. (B) Expression of exogenous *ste11* ^+^ rescued the mating inefficiency of *rhn1*Δ cells. The *rhn1*Δ cells that harbored either pREP1 (mock) or pREP1-*ste11* ^+^ (*ste11* ^+^) vector were examined for mating after incubation on PMG-leu plates at 26°C for 3 days. “n” indicates the number of cells counted.

Phosphorylation of Ser-2 in the Pol II CTD, as well as Lsg1, contributes to Ste11 expression in response to meiotic induction [23]–[25]. Interestingly, we found that Lsk1, which physically binds to Lsg1 and regulates Lsg1 localization [30], suppressed three Rhn1-regulated meiotic genes in vegetative cells. We then investigate whether Rhn1 was required for *ste11* ^+^ induction upon nitrogen starvation. RT-PCR analyses demonstrated that the level of *ste11* ^+^ transcript induced in *rhn1*Δ cells was relatively lower than that induced in wild-type cells (Figure 7A). Moreover, the expression of exogenous *ste11* ^+^ enhanced the mating efficiency of homothallic *rhn1*Δ cells (Figure 7B). Together, these findings indicated that, upon the initiation of meiotic processes, Rhn1 was involved in proper *ste11* ^+^ expression and that the reduced mating efficiency observed in *rhn1*Δ results from the incomplete induction of *ste11* ^+^.

Expression of *ste11* ^+^ in response to nitrogen starvation depends on phosphorylation of Ser-2 in the Pol II CTD, and this phosphorylation is mediated by the Lsk1-Lsg1 CTD kinase complex, Wis1 (a MAP kinase kinase), and Sty1 (a MAP kinase) [23], [24], [30]. Since Rhn1 also contributes to *ste11* ^+^ expression during meiosis, we hypothesize that Rhn1 promotes *ste11* ^+^ transcription or maintains CTD Ser-2 phosphorylation via binding to the Ser-2 modification. Further analyses are required to test these hypotheses.

### Knockdown of *cids-2* by RNAi results in elevated expression of the germline-specific gene *pgl-1* in *C. elegans*

**Figure 8:**
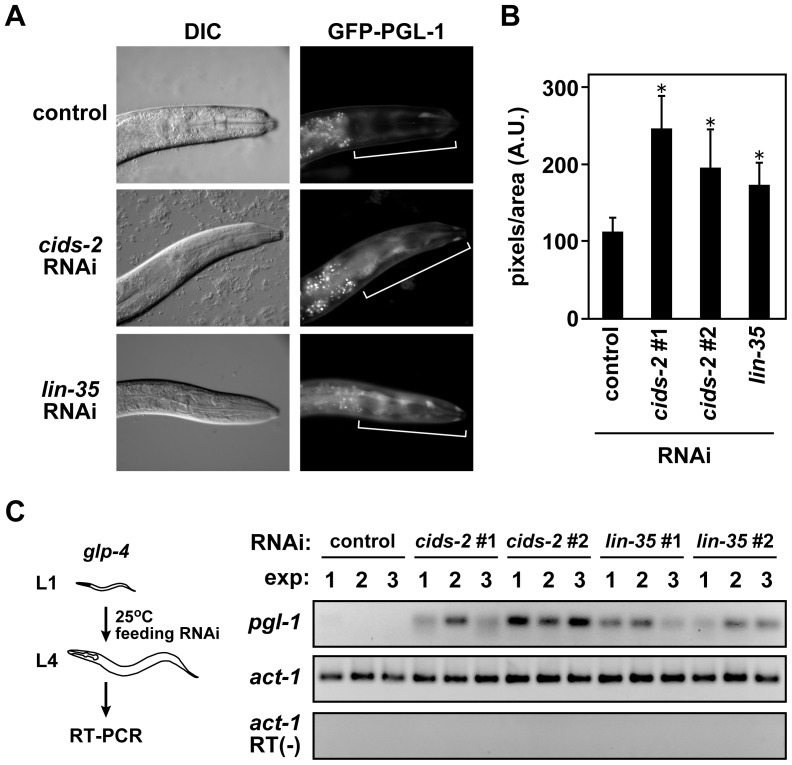
Cids-2 downregulates the expression of PGL-1 in *C. elegans* somatic cells. (A) Differential interference contrast (DIC, left raw) and fluorescent (right raw) images of *C. elegans* (*bnIs1*[*pie-1p::gfp::pgl-1*]) fed *E. coli*. that carry a plasmid for control, *cids-2*, or *lin-35* RNAi. GFP signals in the head regions (white brackets) were elevated in *cids-2* and *lin-35* RNAi worms. (B) The bar graph shows the calculated signal intensity of control, *cids-2*, or *lin-35* RNAi (mean ± S.D., arbitrary units). The GFP signals of *cids-2* and *lin-35* RNAi animals were significantly increased compared to control RNAi worms. *p<0.001, compared to control RNAi. (C) Knockdown of *cids-2* led to the accumulation of the authentic *pgl-1* transcript. (left) A schema of *pgl-1* RT-PCR analysis. Synchronized *glp-4(bn2)* mutant worms at L1 stage were fed *E. coli*. that carried a plasmid for control, *cids-2*, or *lin-35* RNAi and were grown to the L4 stage at the restrictive temperature (25°C). RNAs were then prepared from these L4 animals and subjected to RT-PCR. (right) RT-PCR analysis of the endogenous *pgl-1* mRNA was performed with three independent samples for each of the RNAi-treated groups, and *act-1* was used as a control. RT (-), no reverse transcription.

Meiotic mRNAs are suppressed in vegetative fission yeast, and aberrant inactivation of the meiotic mRNA elimination system, ectopic activation of positive regulators of meiosis, or both result in ectopic of meiosis-specific mRNAs in mitotic cells [33]. We previously suggested that meiotic mRNA elimination may be evolutionarily conserved, in particular in the nematode *C. elegans* [15], because a similar phenomenon, called “soma-to-germline transformation” has been reported [35]–[37]. A BLAST search showed that *C. elegans cids-1* and *cids-2* were most closely related to *rhn1* ^+^. Both *cids-1* and *cids-2* encode Rtt103-related proteins and may play roles in 3′-end cleavage of pre-mRNAs [38]. To examine whether these putative 3′-end cleavage factors might have roles in suppression of germline-specific transcripts, we performed *cids-2* knockdown by RNAi feeding (see Materials and Methods). We chose Cids-2 because Rhn1 was more similar to Cids-2 than to Cids-1. We monitored *gfp::pgl-1* expression from the *pie-1* promoter, which is predominantly active in germline cells, in *cids-2* RNAi animals. We found that *cids-2* RNAi resulted in higher expression of *gfp::pgl-1* in hypodermal somatic cells, including the head hypodermis of adult worms, than did control RNAi (Figure 8A). We quantified GFP signals in the head regions, and the intensity of GFP in *cids-2* RNAi worms was significantly higher than that in control RNAi worms (Figure 8B). The increased GFP signal was specific to *cids-2* RNAi; two independent RNAi constructs targeting different *cids-2* regions resulted in the same phenotype (Figure 8B and data not shown). The increase in *gfp::pgl-1* expression resulting from *cids-2* knockdown was similar to that resulting from knockdown of *lin-35*, which is essential for preventing soma-to-germ transformation [37]. To further confirm the role of Cids-2 in the suppression of germ-specific transcripts, we performed *cids-2* RNAi in *glp-4(bn2)* mutant worms (Figure 8C, left); this mutation has been widely used to generate germline-deficient animals and to assess germline versus somatic gene expression [39]. RT-PCR analysis indicated that the endogenous *pgl-1* transcripts accumulated to abnormally high levels in germline-deficient worms in which *cids-2* RNAi or *lin-35* RNAi was performed (Figure 8C, right panels). These results indicated that meiotic mRNA suppression by Rhn1-related proteins might be conserved in fission yeast and worms.

Soma-to-germline transformation in wild-type *C. elegans* is normally prevented by the Mi-2 complex (MEP-1, LET-418, and HDA-1), the Rb pathway (LIN-35, HPL-1, and DPL-1), and the insulin-like signaling pathway (DAF-2 and AGE-1) [35]–[37]. Our data indicate that 3′-end cleavage factors also contribute to the suppression of soma-to-germ transformation and that the meiotic mRNA suppression system found in fission yeast might be conserved in multicellular organisms. It would be of great interest to investigate whether selective suppression of certain kinds of mRNAs prevents differentiation in mammals.

## Materials and Methods

### Fission yeast strains, media, plasmids, and imaging

The general genetic methods used in this study were as previously described [40]. Complete medium (YEA), minimal medium (PMG), PMG without uracil (PMG-ura), PMG lacking leucine (PMG-leu), and nitrogen-free PMG (PMG-N) were used to culture the cells [40], [41].

A strain carrying the *ura4* ^+^-DSR marker in a wild-type background was provided by Dr. M. Yamamoto (University of Tokyo), and the parental wild-type strain was obtained from the National BioResource Project of Japan (NBRP) at Osaka City University. Other strains expressing a fusion protein with an mCherry, tdTomato [20], GFP [21], PK, or myc epitope-tag and the deletion strains were constructed using PCR-based methods as described previously [42]. All the strains used in this study are listed in Table S3.

Two plasmids, pREP1 and pREP1-*ste11* ^+^, were obtained from Dr. M. Yamamoto and NBRP, respectively. The mating efficiency of each strain was calculated as described [15]. For *ste11* ^+^ expression in *rhn1*Δ, we did not use media that lacked thiamine because the thiamine-repressive *nmt1* promoter of pREP1 was strong; therefore, the basal level of *ste11* ^+^ expression under repressed condition was enough to rescue the mating efficiency. We also found that *ste11* ^+^ induction caused by depleting thiamine was toxic to *rhn1*Δ cells.

An Axio Imager M1 microscope (Carl Zeiss MicroImaging) was used for fluorescence microscopy. The raw images were processed using AxioVision software (Carl Zeiss MicroImaging).

### Immunoprecipitation and western blotting

Immunoprecipitation and western blotting were performed as previously described [15]. Anti-PK (V5005, Nacalai Tesque) and anti-Myc (A-14, Santa Cruz Biotechnology, or 9E10, Covance) antibodies were used in this study.

### RNA analyses of fission yeast

Total RNA samples were prepared from exponentially growing yeast cells in complete or minimal media using the *mir*Vana™ miRNA isolation kit (Life Technologies). DNA contamination in the RNA samples was removed using a TURBO DNA-*free*™ kit (Life Technologies). The DNase-treated total RNA samples were then subjected to RT-PCR using the PrimeScript® II 1st strand cDNA synthesis kit (TaKaRa Bio Inc.) and *TaKaRa ExTaq*® (TaKaRa Bio Inc.). Quantification of RT-PCR results was performed using Quantity One® software (Bio-Rad Laboratories, Inc.)

For microarray analyses, the *S. pombe* expression 4x72K array (090408 Spom exp X4, Roche-NimbleGen) was used. The microarray analyses were independently preformed twice according to the manufacturer's protocol. The data from wild-type cells were compared with those from *rhn1*Δ cells; the average in the fold changes from two independent experiments were calculated, and the transcripts that showed more than a 1.6-fold increase or a 0.5-fold decrease were selected as “increased” or “decreased” mRNAs, respectively. The lists of increased and decreased genes are shown in Tables S1 and S2, respectively. The statistical significance of overlap of two groups was determined using a calculation program (http://nemates.org/MA/progs/overlap_stats.html).

### Experiments with Nematode

*C. elegans* strains were grown under standard conditions [43]. The *C. elegans* strains used in this study were wild-type Bristol N2, SS104 *glp-4(bn2)* [39], and SS747 *bnIs1*[*pie-1p::gfp::pgl-1*] [44]. Gene knockdown was achieved by feeding animals RNAi as described previously [45]. The plasmid vector used for RNAi feeding, pPD129.36 [45], was provided by Dr. A. Fire (Stanford University). To generate RNAi vectors for *cids-2*, two PCR fragments corresponding to the *cids-2* open reading frame (nucleotide positions 86–977 and 1010–1900) were amplified by PCR and then subcloned into pPD129.36, resulting in L4440-*cids-2*#1 and L4440-*cids-2*#2, respectively. We also generated pPD129.36-*lin-35*#1 (nucleotide position 58–916) and pPD129.36-*lin-35*#2 (nucleotide position 1690–2568) for knockdown of *lin-35*.

For the feeding RNAi experiments, *E. coli* carrying empty pPD129.36, L4440-*cids-2*#1, L4440-*cids-2*#2, pPD129.36-*lin-35*#1, or pPD129.36-*lin-35* #2 were fed to synchronized L1 lavae of the transgenic strain *bnIs1*[*pie-1p::gfp::pgl-1*] or *glp-4(bn2)*. The GFP::PGL-1 signal was observed using AxioPlan2 (Carl Zeiss MicroImaging) with a Coolsnap HQ CCD camera (Princeton Instruments). Quantification of the GFP signals was performed using the Image J software (NIH), and the Student's *t*-test was used to determine statistical significance.

For the RT-PCR analyses shown in Figure 8C, the *glp-4(bn2)* worms, which have a temperature-sensitive sterile phenotype, were maintained at 15°C, but were shifted to 25°C as synchronized L1s, which is when feeding RNAi was initiated. Total RNA was extracted from RNAi-induced synchronized *glp-4(bn2)* L4 worms using the RNAiso Plus reagent (TaKaRa Bio Inc.). Reverse transcription and the subsequent PCR were performed using ReverTra Ace® with gDNA remover (TOYOBO Co., Ltd.) and a Thunderbird™ SYBR® qPCR kit (TOYOBO Co., Ltd.), respectively. The PCR primers used to amplify *pgl-1* and *act-1* were previously described [46], [47].

## Supporting Information

Table S1 **The genes upregulated (>1.6) in** ***rhn1*** **Δ.**
(XLS)

Table S2 **The genes downregulated (<0.5) in** ***rhn1*** **Δ.**
(XLS)

Table S3 ***S. pombe*** **strains used in this study.**
(XLS)
